# Just for Chinese people’s stood up—Prof. Fangzhou Gu, father of “sugar pills”

**DOI:** 10.1007/s13238-019-00669-7

**Published:** 2019-12-17

**Authors:** Jun Wu, Dexiang Dong, Ying Li

**Affiliations:** Institute of Medical Biology, Chinese Academy of Medical Sciences, Kunming, 650118 China

**Figure 1:**
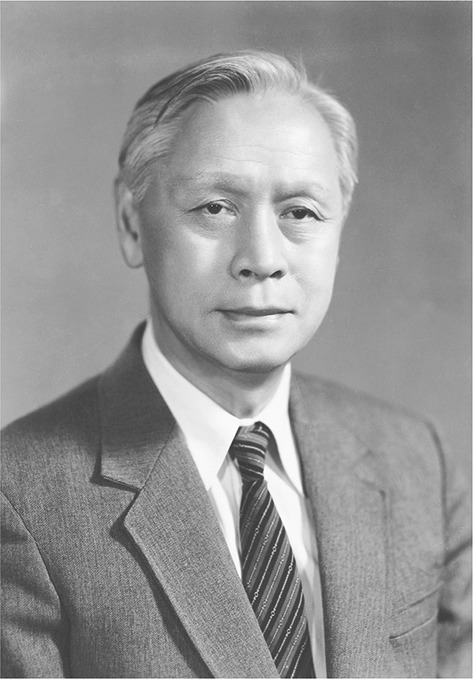
**Professor Fangzhou Gu (1926.6.16–2019.1.2)**

Professor Fangzhou Gu (顾方舟) was born in Shanghai in June 1926. He is a medical scientist, expert virologist, member of the Third World Academy of Sciences, the former president and first-level professor of Peking Union Medical College of Chinese Academy of Medical Sciences. On September 17, 2019, Professor Fangzhou Gu was awarded national medals and honorary titles together with 42 people and he was conferred the “People’s Scientist” national honorary title (Fig. 1).

The family tragedies of his childhood and the humiliating life as conquered people had a tremendous impact on Professor Fangzhou Gu, including his thoughts and national sentiments. He was independent, self-support and hard working. In 1944, Professor Fangzhou Gu was admitted to the Medical School of Peking University and inspired to be a doctor to rescue the people in suffering. After graduating in 1950, he resolutely chose to devote to public health to benefit more people. In October 1950, with such a belief, he readily accepted the school’s assignment and worked at the Dalian Health Research Institute. Since then, Professor Fangzhou Gu developed a deep infatuation for virology.

**Figure 2:**
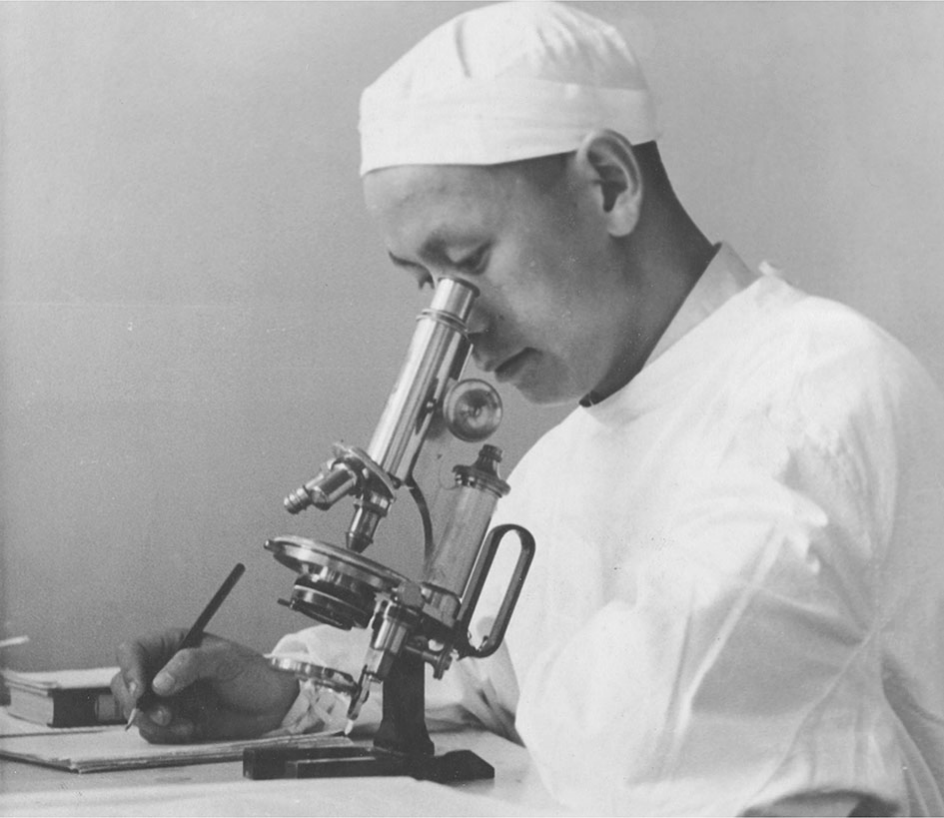
**Professor Fangzhou Gu studied in the Soviet Union**

In August 1951, Professor Fangzhou Gu, as one of the first batch of overseas students who were sent to the Soviet Union after the establishment of the People’s Republic of China, was assigned to the Institute of Virology of the Academy of Medical Sciences of the Soviet Union (Fig. 2).

**Figure 3:**
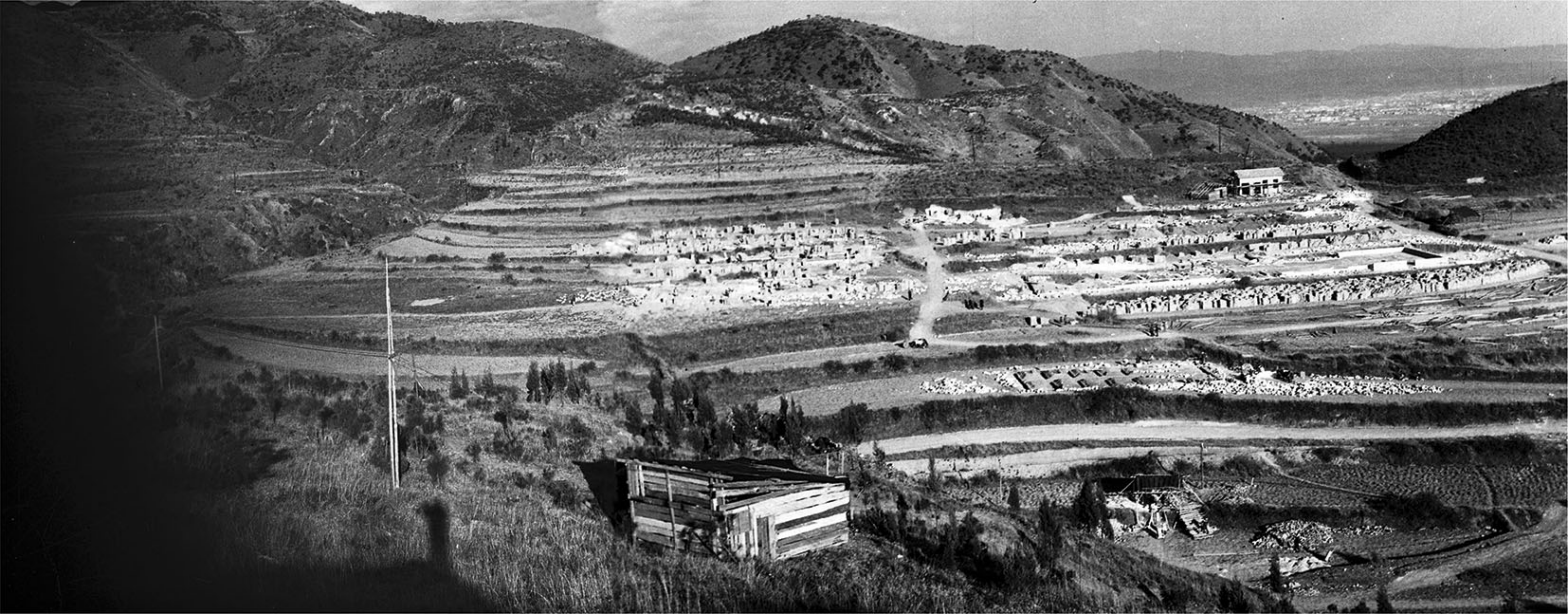
**The Institute of Medical Biology of the Chinese Academy of Medical Sciences was established in the early stage**

Four years of study laid a solid foundation for his later scientific research. After returning home, Professor Fangzhou Gu, at the age of 29, was appointed as the Deputy Head of the Encephalitis Laboratory of the Institute of Microbiology and Epidemiology of the Ministry of Health. In 1958, he was dispatched to join the Institute of Virology of the Chinese Academy of Medical Sciences as the Head of the Polio Laboratory. Since then, he has devoted his whole life into the battles to eradicate this terrible acute viral infection in children. In order to use higher primates in medical research and develop polio vaccine, Qizhen Shen (沈其震), Vice President of the Chinese Academy of Medical Sciences, and Professor Fangzhou Gu went to Yunnan to find a place for Institute of Medical Biology in 1958. On the barren hillside of the Jade Mountain in the western suburbs of Kunming, where no water, no electricity, no roads was connected, the Institute of Medical Biology was built from scratch (Fig. 3).

**Figure 4:**
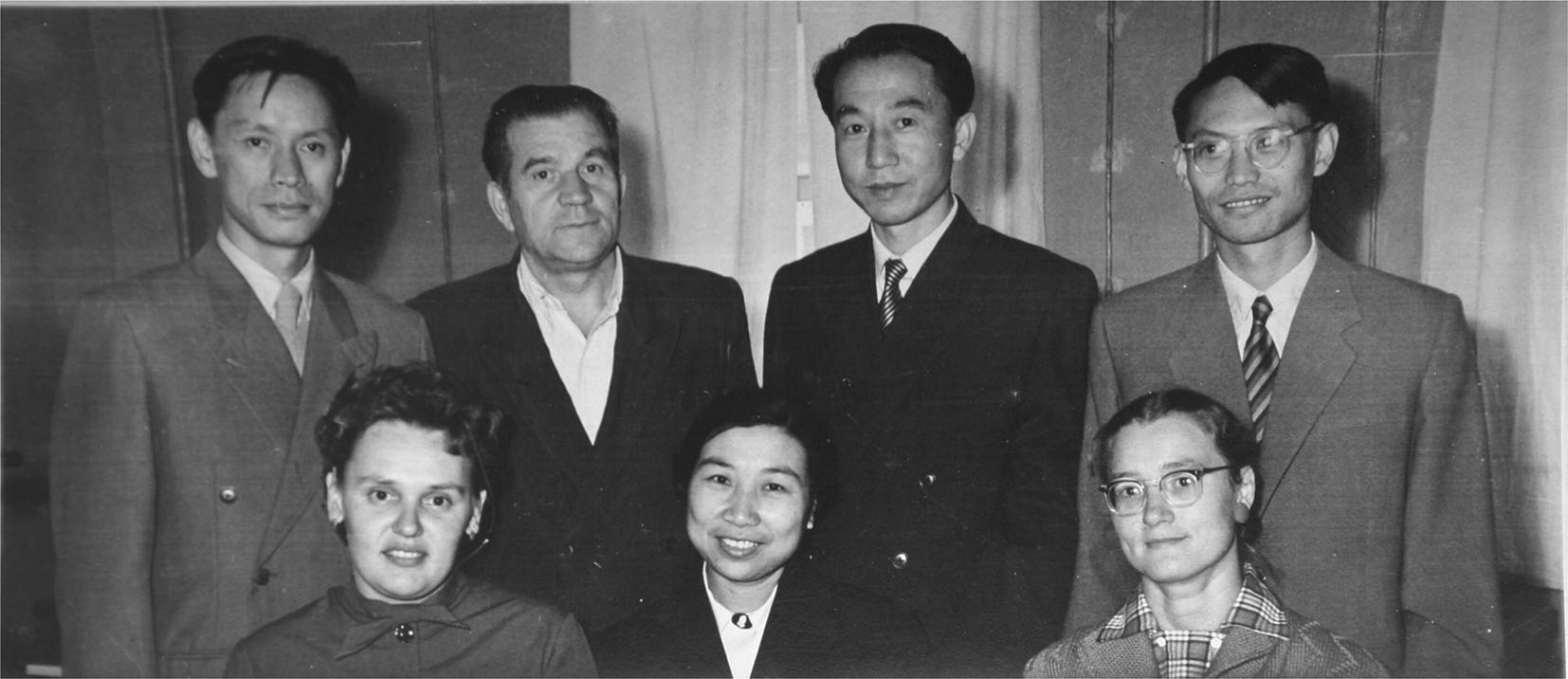
**Professor Fangzhou Gu (The first from left of the second row) and his colleague investigated the vaccine production in Soviet Union**

After the epidemic of polio in Nantong in 1953, the Ministry of Health listed the disease as a legally reported infectious disease. Since then, the epidemic reports have been increasing and the infected area of the disease has been expanding, severely affecting children’s health. In March 1959, the four-member group (Fangzhou Gu, Dexiang Dong (董德祥), Zhongquan Wen (闻仲权), and Jingwu Jiang (蒋竞武)) led by Professor Fangzhou Gu was sent to the Soviet Union to investigate the vaccine production. Professor Fangzhou Gu learned from the academic conference that the United States and the Soviet Union are cooperating to develop a live vaccine for polio (Fig. 4).

He reviewed all the public available materials and compared the advantages and disadvantages between the live and inactivated Polio vaccines. According to the specific situation on China’s huge population and underdeveloped economics at that time, he boldly proposed the strategy of employing the route of the live vaccine. This proposal was approved and supported by the Ministry of Health. This proposal was decisive meaningful for the elimination of polio in China eventually, which also becomes the greatest contribution made by Professor Fangzhou Gu in his life.

Therefore, Professor Fangzhou Gu immediately returned to China and started the development of a live vaccine for polio. In December 1959, the Ministry of Health called for a collaborative team of the Chinese Academy of Medical Sciences, the Beijing Institute and Chengdu Institute of Biological Products to conduct trial production. The collaborative team was again chaired by Professor Fangzhou Gu and conducted at the Beijing Institute of Biological Products. In the early 1960s, the collaborative team used the original attenuated strains, Sabin I, II and III, to produce the very first I, II and III attenuated vaccines for 5 million people in China (Gu et al. 1961). At this time, three phases of clinical trials were required. In the phase I trial, 10 susceptible children were required for safety observation. Professor Fangzhou Gu took the lead role to use the test vaccine on his newborn son, which inspired the other colleagues in the laboratory to participate in the clinical trial. Thus the safety of the vaccine has been successfully verified. The phase III of the clinical trial was mainly for testing the epidemiological effect. In cooperation with the health and epidemic prevention stations in 11 cities including Beijing, Shanghai and Qingdao, nearly 4 million children under the age of 7 were tested for vaccines. The results significantly reduced the incidence and flattened the season peaks. The results of phase III clinical trials demonstrated that live vaccines were safe and effective, with good immunological and epidemiological effects.

In 1961, the collaborative team continued to build a professional team to go to the Institute of Medical Biology in Kunming to expand the scale of trial production and establish the manufacturing base. A four-person team led by Professor Fangzhou Gu was regarded as the backbone scientists, through the training of young technicians to master the technical operations, the production and quality-control instructions and detailed operations procedures were compiled upon the technics of Soviet Union further with the consideration of Chinese situation, which guaranteed the safe production and quality control of the vaccine.

After the decision on choosing the route of live vaccine immunization in 1959, due to the strict requirements for low temperature of the live vaccine, it is necessary to improve the dosage form to ensure the quality of the vaccine. In 1960, Professor Fangzhou Gu proposed to develop a “sugar pill” vaccine, and Dexiang Dong was responsible for the implementation of this project specifically. Qizhen Shen, vice President of the Academy of Medical Sciences, selected Shanghai Xinyi Pharmaceutical Factory to collaborate with Institute of Medical Biology in person, and they used the preparation technology of traditional Chinese medicine pill to wrap the viral liquid in the sugar pill to make a “sugar pill” vaccine. After hundreds of repeated experiments in three years, the “sugar pill” formula and the preparation technology were continuously improved. Finally, the “sugar pill” vaccine which can prolong the shelf life at room temperature and 4–8 °C was successfully produced in 1963 (Dong et al. 1965). In 1964, the vaccine was spread in the whole country and was welcomed by the majority of personnel in epidemic prevention, parents and children, becoming a new powerful weapon for the prevention, control and elimination of polio.

In 1962, the professional team of vaccine production returned to their original organizations after completing the expansion of trial production. In 1963, Dexiang Dong was dispatched back to the Institute of Medical Biology to be responsible for vaccine production. In 1964, Professor Fangzhou Gu was appointed to the deputy director of the Institute of Medical Biology, and his family moved to Kunming. With the growing demand for vaccines, the task of vaccine production continued to increase year by year, from an initial 5 million to 6–70 million per year, up to more than 100 million. The more experience was obtained, the greater improvement on vaccine quality was reached through the revision on production and quality-control instruction every one or two year.

Under supervision of Professor Fangzhou Gu, the Institute of Medical Biology has always adhered to the principle of integration of research, development and utilization, and prompt live vaccines to control and eliminate polio indeed. Every year, scientists and technicians were organized to conduct investigations of virology, serology and epidemics all over the country. They were timely informed of the dynamics of viral transmission, immunological efficacy and existing problems in order to provide scientific basis for continuous improvement of immunization methods, revision of immunization strategies, and implementation of planned immunization. To improve the immune efficacy of each type of vaccines and reduce the number of inoculations, after years of repeated trials and adjustment of the dosage ratio of the types, the trivalent vaccine in sugar pill was successfully developed. In order to encourage the epidemic prevention stations in various provinces, cities and municipalities to actively participate in the investigations on immune effect and epidemiology, with the support of the Ministry of Health, “Experience Exchange Conference of National Prevention and Control of Polio” were organized in 1971, 1975, and 1978. The experience of Polio prevention was spread and planned immunization was promoted; also, a few training courses on polio and other enterovirus experimental technologies were provided to promote virology and serology surveillance.

In 1971, Professor Fangzhou Gu relocated to join the Chinese Academy of Medical Sciences for his work. However, he always paid close attention to the production, utilization and existing problems of live polio vaccines, and often gave his guidance and help. Professor Dexiang Dong recalled that every time he went to Beijing, Professor Fangzhou Gu would inquire and discuss all details of research of polio.

In 2000, the World Health Organization confirmed that China had successfully blocked the spread of native wild poliovirus strains and achieved the goal of eliminating polio. As one of the representatives, Professor Fangzhou Gu signed his own name on the “Certification Report for Eliminating Polio in China” and realized his lifelong dream. This is another pioneering work in the history of public health in the world after the global eradication of smallpox. Fangzhou Gu, as the pioneer initiative in China, has made an invaluable contribution to this work. He dedicated his life to fulfilling his duty and the national mission. Professor Fangzhou Gu will be remembered in people’s hearts forever.

In the memoir of Professor Fangzhou Gu, he wrote as:

*I regard as import what have I done for the people rather than what can I obtain from them all through my life*.

This is also the promise claimed by all the health guardians, never fading with the passage of time. We will succeed and continue the ideal of Professor Fangzhou Gu, safeguard public health and public health safety, and stay true to the medical mission.
